# Quality of life evaluation of children with sleep bruxism

**DOI:** 10.1186/1472-6831-10-16

**Published:** 2010-06-14

**Authors:** Paula M Castelo, Taís S Barbosa, Maria Beatriz D Gavião

**Affiliations:** 1 Department of Biological Sciences - Federal University of São Paulo, R. Artur Riedel, 275, Diadema - SP, postal code 09972-270, Brazil; 2 Department of Pediatric Dentistry - Piracicaba Dental School - State University of Campinas, Av. Limeira, 901, Piracicaba - SP, postal code 13414-903, Brazil

## Abstract

**Background:**

The study of potential factors associated with sleep bruxism (SB) may help in determining the etiology of such parafunction. Thus, this study aimed to evaluate the quality of life (QoL) of children with SB by means of a generic scale, in addition to the association of sociodemographic characteristics and other parafunctional habits.

**Methods:**

This cross-sectional study included healthy children of both genders, aged 7.18 ± 0.59 years, with (n = 25) and without (n = 69) signs and symptoms of SB. Data were collected in caries-free children from public schools by applying a translated and validated version of the *Autoquestionnaire Qualite de Vie Enfant Image *(AUQUEI), clinical examination and interview with the parents. The psychometric properties evaluated for the scale referred to internal consistency (ceiling and floor effects, Cronbach's Alpha coefficient, Items Correlation Matrix, and corrected Item-Total Correlation) and the discriminant validity (*t*-test). By means of logistic regression with stepwise backward elimination, associations were evaluated between SB and age, gender, body mass index, maternal use of alcohol/tobacco/medicine during pregnancy, maternal age at birth, parent's schooling, presence of sucking habit, nail biting, enuresis, number of children, child's order (first born), occurrence of divorce/parent's death, and AUQUEI scores.

**Results:**

The results of the AUQUEI psychometric analysis showed homogeneity of items and a Cronbach's alpha coefficient of 0.65; no negative correlations between the items were found. The mean AUQUEI scores for children with SB did not differ significantly from those of children without the parafunction. Only the independent variable "maternal age at birth" showed a significant negative association with SB.

**Conclusions:**

In the studied sample, children with SB presented scores of QoL that were similar to those without the parafunction, and children from the youngest mothers were more likely to present SB.

## Background

Emotional disturbances have classically been considered to be involved in the etiopathogenesis of parafunctions such as bruxism and nail biting, persistence of sucking habits (bottle feeding, pacifier, digital and labial sucking) and parasomnias (enuresis nocturna). Bruxism consists of a stereotyped movement disorder characterized by grinding or clenching of the teeth and can occur during sleep (sleep bruxism, SB) as well as during wakefulness. According to the *International Classification of Sleep Disorders, second edition (ICSD-2)*, SB is listed in the new sleep-related movement disorders category, and is defined as "an oral parafunction characterized by grinding or clenching of the teeth during sleep that is associated with an excessive (intense) sleep arousal activity" [1,2]. SB is a common condition and there is a great amount of research on its prevalence, effect and management. But the etiology of SB is not well understood to date.

The value of obtaining self reports from children and adolescents about their health, functioning, abilities and emotions is increasingly recognized within both medical care and child health research [3]. The *Autoquestionnaire Qualité de Vie Enfant Imagé *(AUQUEI) is a generic instrument that intends to measure all dimensions of health-related quality of life, and can therefore be applied to healthy populations as well as any clinical population regardless of the type of medical condition. It has been validated and translated for the Brazilian population by Assumpção Jr. et al. [4]. In previous studies, this instrument demonstrated adequate psychometric properties and was sufficiently sensitive to distinguish sick children from healthy ones [4-9].

The study of factors potentially associated with SB could contribute to a better understanding of the nature of this condition and, therefore, might be useful to prevent the development of SB. To our knowledge, the quality of life (QoL) of children with SB has not been investigated; thus, the aim of this study was to evaluate the QoL of children with SB from public schools of Piracicaba (SP, Brazil), and its association with sociodemographic characteristics and other parafunctional habits.

## Methods

### Anamnesis and clinical examination

A cross-sectional study design was used with subjects recruited as a convenience sample from public schools of Piracicaba, SP, Brazil. Five hundred children aged 6-8 years of both genders were evaluated; from this initial sample, ninety-four healthy subjects (mean 7.18 ± 0.59 years) were selected after the conduction of a complete anamnesis and clinical examination in order to verify their medical and dental history, as well as sociodemographic data and body variables (weight and height). The inclusion criteria were the presence of mixed dentition with first permanent molars erupted, no history of trauma, and no previous orthodontic treatment. The exclusion criteria were: dental caries, early tooth loss, systemic and/or mental developmental disorders (including diseases of the endocrine and metabolic systems) and use of medications that could interfere with the central nervous system.

The interviews were conducted using a prestructured questionnaire and data were collected directly from mothers/caregivers. Gender, number of children, birth order of the infants, maternal age, parents' employment and education, marital status at birth, mode of delivery, intention to breastfeed, months of maternity leave, presence and duration of exclusive and non-exclusive breastfeeding and bottle feeding, pacifier use, digital sucking, parafunctional habits (nail biting, SB and enuresis nocturna), and parental death and/or divorce were recorded by the same researcher. Exclusive breastfeeding was defined as breast milk consumption with no supplementation of any type of food or drink (no water, no juice, no non-human milk and no solids), except for vitamins, minerals, and medications [10,11]. Enuresis nocturna was considered when present at least once per month [12].

Finally, the signs and symptoms of SB were recorded, taking into account the following parameters [13,14]:

1. sibling or parental report of grinding sounds (at least three times a week);

2. presence of shiny and polish facets on incisors and/or first permanent molars (based primarily on palatal surface and incisal edges and working cusps, respectively) observed in clinical examination, taking into account the time of eruption;

3. no other medical, mental or sleep disorders (e.g., epilepsy, obstructive apnea syndrome).

Wear facets on deciduous teeth were not considered. The presence of SB was confirmed by both the parental report and the presence of tooth wear, since the latter is a cumulative sign. All clinical examinations were done by the first author (PMC). Kappa statistics were used to evaluate the consistency of intra-examiner reproducibility for SB clinical diagnosis, and the value obtained was 0.78 (substantial agreement). The Ethical Committee of the Dental School of Piracicaba approved the study, and all children and their parents/guardians gave verbal and written permission to carry out the research (protocol n. 021/2007).

### AUQUEI - Autoquestionnaire Qualité de Vie Enfant Imagé

The children's perceptions about their QoL were assessed by applying a translated and validated version of the *Autoquestionnaire Qualité de Vie Enfant Imagé *(Portuguese version of AUQUEI) [4,15]. The AUQUEI consists of a structured-format scale with 26 items, and explores domains of family, autonomy, function, and leisure. The response level for each question (e.g., "how do you feel when having dinner with your family?") was measured by marking faces expressing different emotional states, i.e., very happy, happy, unhappy or very unhappy; these states received scores of 0 to 3, respectively. The cut-off point was established previously [4] at 48; lower scores indicate that the QoL is negatively affected. In addition to the structured scale, the instrument includes an open question in the first part, where the child must call on his/her own experience to calibrate the questionnaire with his/her own standards of satisfaction [15]. The interviews were done by one examiner in a separate room without parent/caregiver presence.

### Statistical Analysis

Statistical analysis was performed using SPSS 9.0 (SPSS, Chicago, IL, USA) with a 5% significance level, and normality was assessed using the Kolmogorov-Smirnov test.

The AUQUEI's psychometric properties were assessed by evaluating ceiling and floor effects, the internal consistency (Cronbach's Alpha coefficient, Items Correlation Matrix, and corrected Item-Total Correlation with Pearson correlation coefficients), and the discriminant validity, i.e., the difference in scores on the AUQUEI and each of its domains between children with and without signs and symptoms of SB (unpaired *t*-test/Mann-Whitney test and Chi-Square test).

A logistic regression model was developed to test the association between SB (as the dependent variable) and quality of life, other parafunctional habits and sociodemographic characteristics. All independent variables were entered into the model, then the least significant terms were regressively dropped until only those with p < 0.05 remained in the model (stepwise backward elimination). The independent variables entered in the model were: age, gender, body mass index, maternal use of alcohol/tobacco/medicine during pregnancy, maternal age at birth, parent's schooling, presence of a sucking habit, nail biting, and enuresis, number of children, child's order (first born), occurrence of divorce/parent's death, and AUQUEI score.

## Results

The main sociodemographic characteristics of the whole sample at birth are shown in Table 1. At birth, the mother's age range was from 16 to 43 years and most of them (83.70%) were either married or living with their partner. The use of alcohol/tobacco/medicine during pregnancy was reported by 32.61%, and the mean exclusive and non-exclusive breastfeeding durations were 3.54 and 9.56 months, respectively. The divorce rate increased from 17.20% at birth to 28.72% at the time of this study. Only one occurrence of the father's death was observed, and one child did not live with either parent.

**Table 1:** Distribution of the sample studied in accordance with sociodemographic and feeding characteristics at birth (n = 94)

	% of the sample	Mean (± SD)	Range
Normal delivery	44.57	-	-
Maternal age at birth (y)	-	26.18 (6.63)	16 - 43
Marital status (married/living with partner)	83.70	-	-
Mother's schooling (8 y or more)	75.00	-	-
Father's schooling (8 y or more)	64.13	-	-
Illness in the first month after birth	20.65	-	-
At least four months maternity leave/did not work	88.04	-	-
Intended breastfeeding duration (months)	-	13.11 (8.48)	3 - 60
Problems during delivery/preterm	10.87	-	-
Report of alcohol/tobacco/medicine use	32.61	-	-
Exclusive breastfeeding duration	-	3.54 (3.41)	0 - 24
Breastfeeding duration	-	9.56 (10.92)	0 - 48

Of 94 subjects selected for this study, 25 (26.60%) presented signs and symptoms of SB. Nail biting was the most frequent parafunction observed (43.61%), and 29.79% presented at least one type of sucking habit (nutritive and/or non-nutritive) (Table 2).

**Table 2:** Distribution of the sample studied in accordance with age, gender, body variables, sociodemographic characteristics, and the presence of parafunctional habits (n = 94)

	Mean	± SD			n	%
Age (y)	7.18	0.59		Bruxism	25	26.60
Weight (Kg)	28.49	7.40		Sucking habit	28	29.79
Height (m)	1.28	0.06		Nail biting	41	43.61
BMI	17.31	3.39		Enuresis nocturna	15	15.96
Number of children	2.33	1.23		Divorce/parent's death	27	28.72
Gender 48 ♀ 46 ♂				First-born	43	45.75

BMI, body mass índex (Kg/m^2^).

The results of the AUQUEI psychometric analysis showed homogeneity of items (Table 3). There were no negative correlations between the items, and the inter-item correlation coefficients among the scores of the four domains were between 0.20 and 0.38. The item-total correlations were between 0.50 and 0.72 (Table 4). A ceiling effect was only present for the domain *leisure *(i.e., above 10%). Cronbach's alpha for the whole scale was 0.65 (indicating acceptable internal consistency), and the alpha level did not increase when any item was deleted. In the discriminant validity analysis, the differences between scores on the AUQUEI and each of its domains were tested between children with and without signs and symptoms of SB. The mean score for children with SB did not differ significantly from that for children without such parafunction (Table 5), even when the cut-off point of the scale was considered.

**Table 3:** Psychometric properties of AUQUEI - internal reliability analysis: Items Correlation Matrix

r (*p-value*)	Function	Family	Leisure	Autonomy
**Function**	-			
**Family**	0.33 (*0.001*)	-		
**Leisure**	0.20 (*0.058*)	0.23 (*0.027*)	-	
**Autonomy**	0.29 (*0.005*)	0.38 (*< 0.001*)	0.22 (*0.037*)	-

r = correlation coefficient

**Table 4:** Psychometric properties of AUQUEI - internal reliability analysis: floor effect, ceiling effect, item-total correlation, Cronbach's Alpha and item-deleted Alpha

Domains	Mean (± SD)	Floor effect*		Ceiling effect^†^		Item-total correlation r *(p-value)*	Alpha if item deleted
		n	%	n	%		
Function (0-15)‡	9.38 (2.18)	0	0.00	0	0.00	0.70 *(< 0.0001)*	0.58
Family (0-15)‡	10.56 (2.53)	0	0.00	4	4.26	0.72 *(< 0.0001)*	0.56
Leisure (0-9)‡	8.06 (1.12)	0	0.00	41	43.62	0.50 *(< 0.0001)*	0.63
Autonomy (0-15)‡	8.37 (2.57)	0	0.00	0	0.00	0.66 *(< 0.0001)*	0.59
Total scale (0-78)‡	53.69 (7.15)	0	0.00	0	0.00	Cronbach's Alpha = 0.65	

‡ interval of possible values

* % of children with score = 0

† % of children with maximum score

**Table 5:** Sample distribution in accordance with quality of life scores and the presence of bruxism

Cut-off point	With bruxism		Without bruxism	
	**n**	**%**	**n**	**%**
	
< 48	5	20.00	14	20.29
≥ 48	20	80.00	55	79.71
Total^†^	25	100.00	69	100.00

Mean score (± SD)^‡^	53.04 (± 6.45)		53.93 (± 7.41)	

† p > 0.05 (Chi-Square Test)

‡ p > 0.05 (unpaired *t*- test)

Backward stepwise logistic regression revealed that lower maternal age at birth led to a significantly higher probability of SB in the studied sample (Table 6). Other parafunctional habits, divorce/parent's loss, parent's schooling, and use of medications/tobacco/alcohol during pregnancy were not associated with SB.

**Table 6:** Backward stepwise logistic regression to test the association of independent variables with sleep bruxism in the studied sample (n = 94)

Dependent variable	Independent variables	Coef.	*p- *value	Odds ratio	95% CI	Significance of the model		
						R	*p-*value	Power
	Constant	0.713	-	-	-			
	Age	-	0.558	-	-			
	BMI	-	0.431	-	-			
	Gender	-	0.993	-	-			
	Maternal age at birth	-0.017	0.015	0.902	0.830-0.980			
	Maternal use alcohol/tobacco/medicine	-	0.258	-	-			
	Mother's schooling	-	0.852	-	-			
Bruxism	Father's schooling	-	0.797	-	-	0.252	0.015	0.687
	Sucking habit	-	0.616	-	-			
	Nail biting	-	0.550	-	-			
	Enuresis	-	0.628	-	-			
	Number of children	-	0.767	-	-			
	First born	-	0.773	-	-			
	Divorce/parent's death	-	0.522	-	-			
	AUQUEI score	-	0.602	-	-			

BMI, body mass index; AUQUEI, Autoquestionnaire Qualite de Vie Enfant Image.

## Discussion

AUQUEI was developed and tested in the French population by Manificat and Dazord [15]; Assumpção Jr. et al. [4] translated and validated the instrument for Brazilian children. It has subsequently been tested for healthy children and ones with disease conditions such as human immunodeficiency virus and/or acquired immunodeficiency syndrome [8], stomas [16], dental caries [6], cerebral palsy [17], and kidney or liver transplantation [18]. The advantage of generic questionnaires is that they provide a comprehensive overview of QoL at the individual or group level [19]. According to Gherunpong et al. [20], the picture aids allow adaptation to suit the children's competence, assist motivation and reduce anxiety. The results showed homogeneity among the items, and all inter-item correlations were positive and not high enough for any item to be redundant. Moreover, all item-total correlations were above the minimum recommended level of 0.20 [21]. The Cronbach's Alpha level reached (0.65) was below the recommended level of 0.70 [21]; however, as cited by Gherunpong et al. [20], alpha is not a perfect indicator of reliability as it tends to underestimate the reliability of multidimensional scales, and lower values can be expected from health-related measures. In the study done by Thöni et al. [8], AUQUEI was useful for evaluating the decline of QoL in HIV-infected children over months of therapy. Thus, QoL evaluation can be used for different groups of subjects, predict future evolution from a prognostic perspective, and evaluate change over time [22]. On the other hand, the generic instrument has the disadvantage that it is not able to provide a detailed and reliable measurement of dimensions that are specific for a certain condition.

Bruxism has a multifactorial etiology and there is no consensus in the literature about the relevance of each factor for its development. Occlusal factors, psychosocial and environmental factors, medicines, stress, and anxiety have been described as bruxism triggers [23]. In this study, the QoL of children with and without SB was investigated using a generic scale, and the instrument did not clearly discriminate between them. Comparable studies that discussed the relationship between QoL and bruxism in children were not found in the literature. However, previous studies reported that anxiety may be a prominent factor in the development of bruxism in children [24,25]; when the anxiety is treated, either with psychological techniques or with drugs, the signs of bruxism may decrease [24,26]. A specific instrument that also explores the child anxiety level may therefore be useful in future studies to understand whether familial, social, emotional and physical factors are related to anxiety traits and SB in young subjects.

The prevalence studies of SB are complicated because of its variability over time and the limitations of the clinical diagnostic criteria. Some helpful diagnostic criteria have been proposed by the American Academy of Sleep Medicine [1], and they consist of anamnestic and clinical indicators and serve as practical descriptors of SB for both clinical and research purposes. Indeed, a confirmed diagnosis is only possible using tools such as audio-video and electrophysiologic recordings, but these methods are expensive and not likely to be used in studies with large numbers of individuals. Thus, a collection of signs and symptoms related to SB in conjunction with complaints by a sibling or parent is still the most efficient and reasonable way to assess SB in a clinical setting [14].

The investigation of potential factors associated with SB in the studied sample showed that lower maternal age at birth led to a significantly higher probability of SB in the childhood. It is therefore important to pay special attention to determining negative parental behaviors related to early pregnancy such rejection, lack of affection and conflict. Moreover, socioeconomical and educational limitations may be present in a non-intended pregnancy. According to Luecken and Lemery [27], impaired early parent-child relationships can not only contribute to the development of negative psychological (e.g., depression, anxiety, hostility) and social characteristics, but also increase risk of the development of physical illness. Of course, given the limitations of this study, these findings should be cautiously interpreted. Parafunctional habits such nutritive and non-nutritive sucking habits and enuresis were not associated with SB. Nail biting, a deleterious habit that may increase when the child feels anxiety, worried or stressed, was also not associated with SB [28,29].

Other demographic variables investigated in this study such as divorce and parent's schooling were not associated with the parafunction. Limitations of this study may include a lack of socioeconomical diversity in the study population, which may affect the familial structure and QoL of the children. Thus, continued research about QoL in children with SB is required for generalization of the results.

## Conclusions

In the studied sample, children with sleep bruxism presented quality of life scores similar to those of children without the parafunction and children of the youngest mothers were more likely to present sleep bruxism.

## Abbreviations

AUQUEI: *Autoquestionnaire Qualite de Vie Enfant Image*; QoL: Quality of life

## Competing interests

The authors declare that they have no competing interests.

## Authors' contributions

PMC was the main author of the present manuscript and she participated in all stages throughout the work. TSB made critical comments on the manuscript. MBDG was the head of the present research and acted as a supervisor. All authors read and approved the final manuscript.

## Author's information

**PMC**: Professor at the Department of Biological Sciences - Federal University of São Paulo, R. Artur Riedel, 275, Diadema - SP, postal code 09972-270, Brazil.

**TSB**: Graduated in the Program of Behavioral-Multidisciplinary Medicine; Post Graduate Student at the Department of Pediatric Dentistry - Piracicaba Dental School - State University of Campinas, Av. Limeira, 901, Piracicaba - SP, postal code 13414-903, Brazil.

**MBDG**: Professor at the Department of Pediatric Dentistry - Piracicaba Dental School - State University of Campinas, Av. Limeira, 901, Piracicaba - SP, postal code 13414-903, Brazil.

## Pre-publication history

The pre-publication history for this paper can be accessed here:

http://www.biomedcentral.com/1472-6831/10/16/prepub
